# Sensitive Fibre-Based Thermoluminescence Detectors for High Resolution *In-Vivo* Dosimetry

**DOI:** 10.1038/srep13309

**Published:** 2015-08-28

**Authors:** Mostafa Ghomeishi, G. Amouzad Mahdiraji, F. R. Mahamd Adikan, N. M. Ung, D. A. Bradley

**Affiliations:** 1 Integrated Lightwave Research Group, Faculty of Engineering, University of Malaya, 50603 Kuala Lumpur, Malaysia; 2 Clinical Oncology Unit, Faculty of Medicine, University of Malaya, 50603 Kuala Lumpur, Malaysia; 3 Department of Physics, University of Surrey, Guildford, GU2 7XH, U.K; 4 Department of Physics, Faculty of Science, University of Malaya, 50603 Kuala Lumpur, Malaysia

## Abstract

With interest in the potential of optical fibres as the basis of next-generation thermoluminescence dosimeters (TLDs), the development of suitable forms of material and their fabrication has become a fast-growing endeavour. Present study focuses on three types of Ge-doped optical fibres with different structural arrangements and/or shapes, namely conventional cylindrical fibre, capillary fibre, and flat fibre, all fabricated using the same optical fibre preform. For doses from 0.5 to 8 Gy, obtained at electron and photon energies, standard thermoluminescence (TL) characteristics of the optical fibres have been the subject of detailed investigation. The results show that in collapsing the capillary fibre into a flat shape, the TL yield is increased by a factor of 5.5, the yield being also some 3.2 times greater than that of the conventional cylindrical fibre fabricated from the same perform. This suggests a means of production of suitably sensitive TLD for *in-vivo* dosimeter applications. Addressing the associated defects generating luminescence from each of the optical fibres, the study encompasses analysis of the TL glow curves, with computerized glow curve deconvolution (CGCD) and 2^nd^ order kinetics.

Since 1985 there has been marked decrease in the use of film dosimeters for personal monitoring, largely being replaced by thermoluminescence dosimeters (TLDs)^1^. *In-vivo* TLD dosimetry has also become prevalent^2^,^3^, as exemplified by the therapeutic level work of White *et al.*^4^, the dosimeters monitoring photon radiation. To this can be added other examples of *in-vivo* studies making use of TLDs as the main dosimetry system, for doses typically ranging from 0.1–10 Gy^3^,^5^,^6^,^7^,^8^ and for experiments performed on the total body, within a similar range of dose^9^. When parts of the body that are particularly radiosensitive have been targeted for radiotherapy (including the eyes, glands, head and neck), the spatial resolution of the TLD becomes that much more important^10^,^11^.

Optical fibres have been shown to be a potential candidate for such radiation dose sensors, with particularly high spatial resolution, linear response over wide range of doses and acceptable sensitivity, the latter at a level that has now become comparable with that of commercially available dosimeter sensors^12^. In addition, optical fibres offer multiple options for dose measurement methods both in the form of offline monitoring, i.e., based on TL and optically stimulated luminescence (OSL), and real-time monitoring, i.e., based on OSL and radioluminescence^13^,^14^. In radiation therapy, a high sensitivity dosimeter would be extremely helpful in precision measurement of dose delivery, both to the tumour as well as in out-of-field measurements, such as potential neutron contributions from accelerators operated at high energies (≥10 MeV) becoming an important consideration^15^. Such performance can be expected to aid in obtaining an improved treatment outcome, in terms of enhanced tumour control and reduced post-radiation therapy complications^16^. Several different materials have been doped in silica glass and optical fibres in an effort to improve the radiation dose sensitivity, including germanium^12^,^17^,^18^,^19^,^20^, lithium and barium^21^, aluminium^17^, zirconium oxide (ZrO_2_)^22^, manganese doped calcium tetraborate (CaB_4_O_7_:Mn) nonocrystal^23^, lithium potassium borate glass doped with titanium oxide (TiO_2_) and magnesium oxide (MgO)^24^.

Recently, together with our collaborators in radiation physics, we have reported on the performance of several tailor-made optical fibre TLDs^25^,^26^,^27^,^28^,^29^,^30^,^31^, initial observations pointing to the considerable potential of flat optical fibres^31^,^32^ as a sensitive TL material for radiotherapy applications^26^,^27^,^29^,^31^. In addition, we have recently compared the performance of a capillary fibre with that of a flat fibre (FF) fabricated from the same fibre preform, confirming significant TL yield improvement from the FF compared to that of capillary fibre^28^,^33^. Conversely, to-date the performance of a FF compared to a conventional cylindrical type fibre fabricated from the same fibre preform has not been demonstrated. In addition, detailed standard radiation dosimeter characterizations of such optical fibres have yet to be reported.

In this study, a Ge-doped optical fibre preform has been fabricated using a modified chemical vapour deposition (MCVD) process, half of the preform being made to collapse during the process, while the other half has been left in an uncollapsed state. Using the collapsed part, the preform has then been drawn down into a conventional cylindrical fibre while the uncollapsed part of the preform has been used to fabricate capillary fibre and FF. Comparison between the three types of optical fibre, all fabricated from the same preform, has been carried out in an effort to elucidate differences in radiation dosimeter characteristics, including in linearity, sensitivity, fading and repeatability. In addition, identification and characterization of the defect centres induced in these fibre types as a result of the fabrication process has been analysed in terms of the associated TL glow curves, providing an essential step in understanding the mechanism of TL. The overall aim of this study is to compare and evaluate performance and characteristics of these three type optical fibres as a potential TLD for high resolution ionizing radiation detection, in particular for *in vivo* radiation therapy and similar applications. To the best of our knowledge, this is the first report of its kind, comparing the response of these fibres with the popular phosphor-based LiF:Mg,Ti TLD, TLD-100.

## Results

### TL response

**Figure 1:**
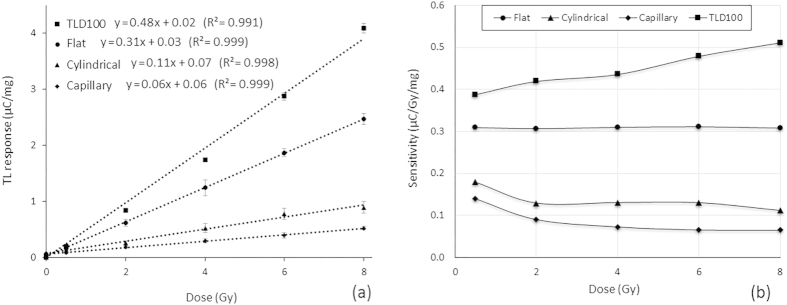
(**a**) Comparison of the TL yield of the three different types of fibre TLD and of TLD-100 using 6 MV photon radiation. The error bars were based on the least square mean value of five different measurements. (**b**) Calculated sensitivities for the three fibre TLD samples compared against that of TLD-100. The flat fibre displays uniform sensitivity across the entire dose range.

Figure 1a shows the TL response of the three types of optical fibres. Over the investigated dose range, a linear TL response has been obtained for all of the optical fibres. Of the three fibre types, the capillary fibre TLD shows the least TL response. On the basis of mean values, the cylindrical fibre provides a TL yield some 1.6 × that of the capillary fibre, while the flat fibre TLD produces a TL yield some 3.2 × and 5.5 × that of the cylindrical and capillary fibre samples respectively. In addition to the variation observed for each data point, the variation in the slope for each dosimeter type has also been calculated based on the maximum and minimum values for each dose and the transitional line across these points [18]. These variations were about 8%, 6%, 10% and 13% (±0.040, ±0.004, ±0.013 and ±0.039) for the TLD-100 (the LiF:Mg,Ti dosimeter), capillary, cylindrical and flat fibre accordingly.

For 6 MV photon radiation Fig. 1b provides sensitivity curves for the various fibre dosimeter samples, compared against that of TLD-100. In accord with expectation, the flat fibre TLD produces the greatest sensitivity of all of the fibre samples, also displaying uniform sensitivity over the full range of measured doses. Conversely, the capillary and cylindrical fibre TLDs are of greater sensitivity at low doses than at the more elevated doses.

### Minimum detectable dose

Minimum detectable dose (MDD) is a particularly important parameter, defining the realm of applicability, not least setting limits on use for low dose irradiation evaluations. The value of MDD depends not only on the medium but also on reader-based parameters including background noise (BG) and photo multiplier tube noise (PMT). The various influences act to define the slope of TL response (α) of the TLD medium and the standard deviation of the background TL signal (*σ*) of the samples. Various determinations of the MDD can be defined, based on the order of the background standard deviation. Here, the criterion of 2*σ* above background is applied in expressing the MDD^34^, as follows:


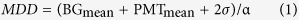


The MDD for the TLD samples, calculated for 6 MV photon irradiation, are shown in Table 1. The average background and PMT noise and its variation measured in this study were 8.98 ± 0.28 nC and 0.45 ± 0.09 nC respectively.

### Energy dependency test

**Figure 2:**
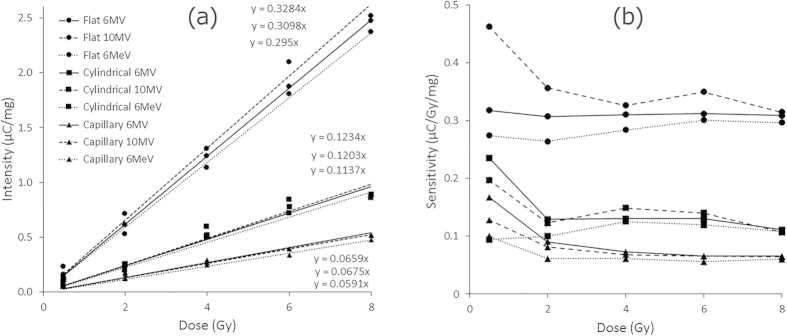
Energy dependency (**a**) and sensitivity (**b**) of the flat, cylindrical and capillary fibre TLD samples for two different photon energies, generated at 6 and 10 MV, and one electron energy, 6 MeV.

Another important detector performance parameter is that of dependency on irradiation energy. The samples were tested for two available photon energies that are regularly used in radiotherapy, 6 and 10 MV, plus one electron energy at 6 MeV. Irradiations were made using five different doses. The results are presented in Fig. 2(a). For each type of TLD sample no significant evidence exists for a difference in response with energy. In regard to the slope for each type, the maximum variation for the different energies was 11%, 8% and 14%, for the flat, cylindrical and capillary fibres respectively. Moreover, the standard deviations of the energy variation were found to be as low as 0.17 for the flat fibre and 0.05 for both the cylindrical and capillary fibres. The slightly energy dependent behavior of optical fiber would be due to their high Z_eff_ number as they are not water equivalent, therefore exhibiting a varying sensitivity to dose response with applied energy. Ideally, a dosimeter should have no or small energy dependence but for dosimeters with relatively significant energy dependence, an accurate reading can still be obtained with correction factors applied appropriately. For example, diode dosimeters, which have large energy-dependence (up to ~30%)^35^, are still being used commercially and widely in radiotherapy dosimetry, subjected as they are to application of correction factors. However, the determination of correction factors for the energy dependence of the proposed fibers is beyond the scope that has been intended of this study.

Figure 2(b) shows the sensitivity of the three optical fibres under the same three energies, of 6 and 10 MV photon and 6 MeV electron irradiation. Almost in all energies and fibre types, a relatively flat sensitivity is observed within 2 to 8 Gy. However, a slight dose dependency towards higher sensitivity is observed at the lower dose of 0.5 Gy for most of the fibres and energies. As has been observed earlier, the flat fibre shows higher dose detection sensitivity compared to the cylindrical and capillary fibres.

### Repeatability test

**Figure 3:**
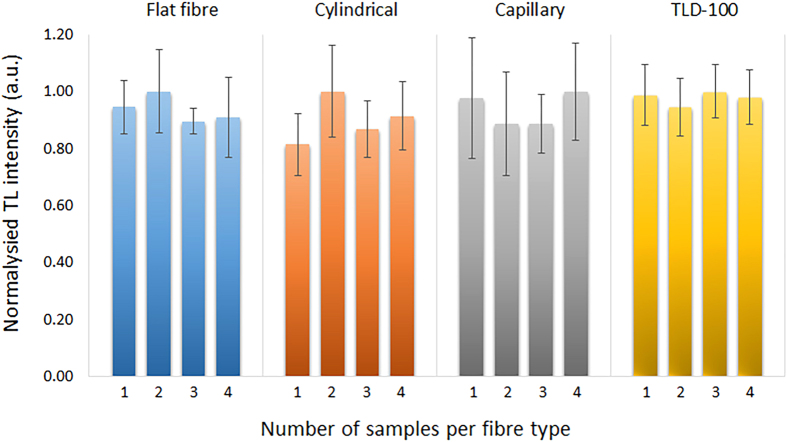
Repeatability test for four different samples from each fibre type reused for 4 complete cycles.

Since it is typical for TLDs to be recalibrated and reused multiple times, a repeatability test has been performed, measuring the variation among four different samples per fibre type over four experimental cycles. This is performed by annealing and re-irradiation followed by read-out of the same fibre samples. Figure 3 shows the normalized TL repeatability variation observed over the four cycles. For flat fibre repeatability the variation range is from 5 to 15% (with an average of 11%) for the four different samples. For the cylindrical fibre, the variation range is 11 to 16% (average of 13%) while for the capillary fibre it is 11 to 19% (average of 16%); by comparison, for TLD-100, the range variation is 10 to 12% (average of 11%). Referring to the average values, flat fibre offered the least variation compared to the other fibres and of highly similar variation compared to the commercial dosimeter, TLD-100. The comparison has been further extended to compare with commercial standard single mode fibres (SSMFs). Results of TL response repeatability test for two different SSMFs fabricated by different manufacturers has been reported^20^ showing an average variation of 11 and 8.7% in the two fibres and similar maximum variation of 13.1% in both SSMFs. These results confirm the repeatability variation of the proposed flat fibre to be comparable to that of other standard fibres and TLD-100.

### Fading analysis

**Figure 4:**
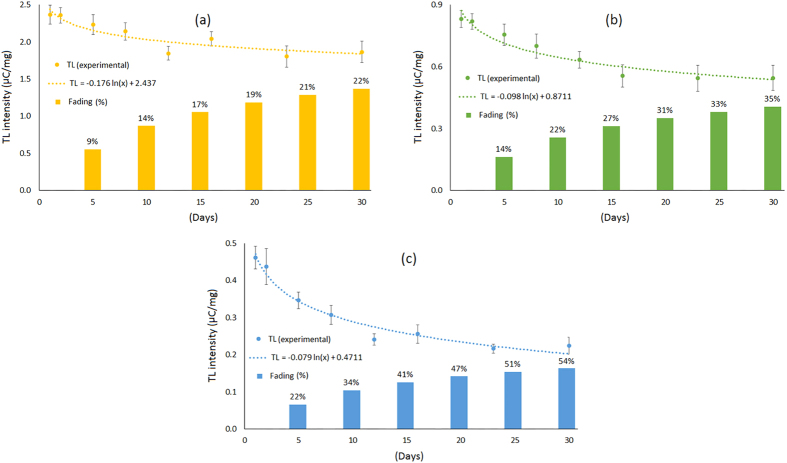
Fading of: (**a**) flat fibre; (**b**) cylindrical fibre, and; (**c**) capillary fibre, all for a period of one month subsequent to irradiation to 6 MV photons to a dose of 8 Gy.

Fading analysis results are shown in Fig. 4a–c, for the flat, cylindrical and capillary fibres respectively. All fibre samples were irradiated simultaneously, use being made of the 6 MV photon source, to a dose of 8 Gy. The TL yield of each set of 5 samples per fibre type were obtained over specified post-irradiation times, from one day to one month post-irradiation. The loss of TL response follows an exponential-like curve, showing rapid fading in the first few days followed by a more linear-like loss subsequently. After 30 days post-irradiation, the TL response for the flat fibre has reduced by 22% compared to that obtained one day after radiation. For the cylindrical and capillary fibres respectively the fading is about 1.6 and 2.5 times greater.

### Glow curve analysis

**Figure 5:**
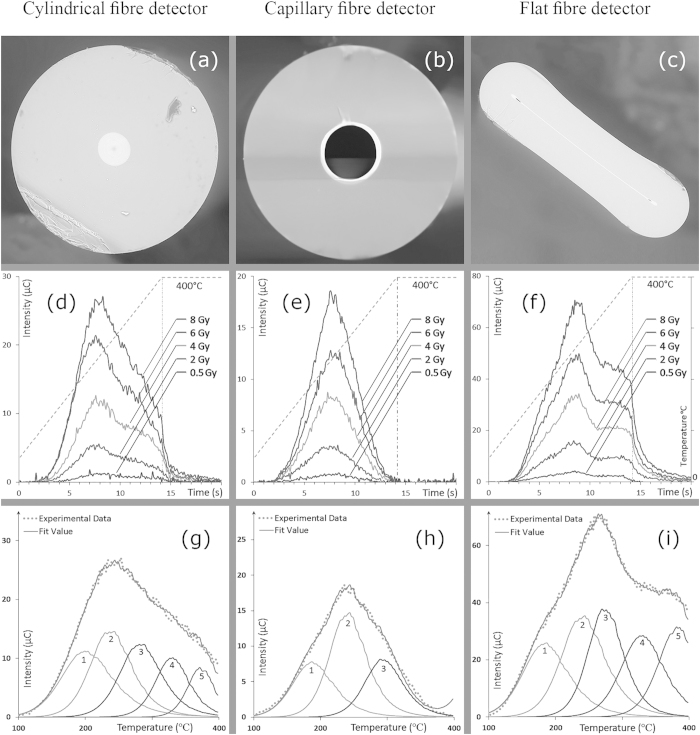
Glow curve analysis. The sub-panels (**a–c**) are SEM cross-sectional images for the cylindrical, capillary and flat fibre, respectively; sub-panels (**d–f**) are the respective glow curves obtained using five different doses, provided in the form of TL intensity versus acquisition time, with temperature represented on the right-hand y-axis, and finally; sub-panels, (**g–i**) show the results of glow curve deconvolution calculated according to second-order kinetics modelling for the cylindrical, capillary and flat fibre respectively.

The distribution of TL peak intensity provides information on the distribution of electron trap energies, moderated by the particular irradiation conditions and heating rate used. Figure 5a–c are respective scanning electron microscope (SEM) cross-section images of cylindrical, capillary and flat fibre samples fabricated for this study. The germanium doped area is the brighter narrow strip within the samples. The TL yield versus readout time continua in Figs 5d–f reveal the uniformity of shape of the glow curves for each of the sample types across the different irradiated doses. Compared with commercial TLDs with thermal luminescence at well-defined temperatures, it is apparent that the fibre TLDs exhibit a broad range of thermal excitation, as expected from an amorphous system. In all optical fibres, the TL glow curves were generated from practically identical time/temperature profiles. Of note are the broad differences between the glow curves of the three types of fibre; the glow curve of the capillary fibre is essentially composed of one centrally located peak producing a maximum intensity value at a temperature of 235 °C (Table 2b), the distribution completing at the maximum temperature of 400 °C while for the cylindrical fibre, a small shoulder is seen to initiate at around 330 °C, growing in intensity with dose (Table 2a), its full intensity artificially terminating as a result of the maximum temperature of 400 °C. The abrupt termination at the maximum temperature set is indicative of deeper defects within the material, incompletely released as a result of the limited activation energy. This same situation is seen with even greater clarity in the case of the flat fibre glow curves, the deeper and more superficial defects being better resolved as a consequence of more pronounced defects localisation. Other than the fact that the TL intensity of the superficial defects peak in the flat fibre is markedly greater than that of the cylindrical fibre, the small shoulder observed in the cylindrical fibre glow curve is now revealed to be a new/secondary glow peak, peaking in intensity at a temperature of around 380 °C (Table 2c). This new source of thermoluminescence in the flat fibre is suggestive of the generation of new defect centres, clearly absent in the capillary fibre. The most apparent outcome of the flat fibre fabrication process is the formation of a rather extensive fused internal surfaces interface, with other aspects of the treatment remaining otherwise common to that of the other fibre types. This suggests that the new defect centres are a result of the fusing of the Ge-doped layer available in the capillary fibre, fused in the cylindrical fibre during the MCVD process and in the flat fibre during fibre drawing process. The latter method shows a significant improvement to be available in defects generation, thought to be due to the strain induced in the collapsed surfaces and the fast quenching rate in the drawing process, essentially freezing in the material before atomic bonds are formed between the joining wall surfaces. Reproducibility of the proposed fibre is realizable by following the same MCVD and fibre drawing parameters. However, showing the detail of reproducibility results is not within the scope of this study.

Figure 5g–i show glow curve deconvolutions for the cylindrical, capillary and flat fibres respectively, computed in accord with second order kinetics, the detailed glow peak parameters being presented in Table 2. For the cylindrical fibre glow curve (Fig. 5g and Table 2a), five main glow peaks are found in which the first three are related to Si-O and Ge-O centres and the last two are Ge structural defects. However, for the capillary fibre glow curve (Fig. 5h), three main glow peaks are found. Based on the simple shape and fabrication process for the capillary fibres, it is expected that these peaks are also representative of Si-O and Ge-O centres in the fibre. Finally in Fig. 5i, the flat fibre glow curve is deconvoluted into five main glow peaks. The first three clearly have the same origins as that of the other two sample types, while the remaining two are Ge structural defects. Although the second peak for both the cylindrical and flat fibres are a result of structural changes, the nature of the peak related defects are expected to be different, with different peak positions (i.e. at different T_max_) and hence different activation energy. More definitively, the first and second peaks, with activation energies less than 1.2 eV are to be related to the existence of O^+^ and O^−^ in the silica network, while the third peak with energy of round 1.2–1.4 eV is to be related to the formation of silicon/germanium nano-clusters in the amorphous silica. The remaining two peaks found within the glow curves for the cylindrical and flat fibre, with energies up to 2.6 eV, are suggested to be due to Ge nano-clusters or ion defects in the fibre^36^.

## Discussion

The results show that while all the optical fibres have been fabricated from the very same Ge-doped preform, their TL response is significantly different (Fig. 1a). Since the TL response derives from the presence of defect centres^37^, present results point to the presence of far greater numbers of defect centres in the flat fibre than in either the cylindrical or capillary fibre. The results further suggest that by collapsing the optical fibre wall surface, an additional different form of defect is generated at the fused internal surfaces. While this defect type is also apparent in the central core of the cylindrical fibre, formed by collapse during the MCVD process, the numbers of defects produced in the flat fibre, formed by collapse during fibre drawing process, is significantly greater. This additional defect is due to the fast quenching rate and strain shearing effects at the fused walls formed during the fibre drawing process.

The results show that like the TLD-100, the flat fibre is linearly sensitive to dose within the investigated dose range, down to doses of 0.5 Gy at a minimum. Conversely, the cylindrical and capillary fibres are seen to show a supralinear response^38^ (Fig. 1b). Further to this, the low dose detection limit of the flat fibre TLD has been shown to be some 30 mGy.mg (Table 1), superior to the performance of any of the other fibre TLD sample types investigated, while it is slightly worse than TLD-100. This points to the flat fibre having potential for low dose detection applications, as in measurements made directly adjacent to the direct radiotherapy field (in other words, out-of-field measurements).

An important characteristic of these fibre TLDs is their relative independence to radiation energy for the therapeutic range investigated herein (Fig. 2). To first order, the TL response can be related directly to the delivered radiation dose and not to the choice of energy, varying on average by no more than 11% across the energies investigated.

In repeatability testing, the flat fibre offered the least variation compared to the other fibres, while its variation was about 3% greater than that of the TLD-100 and 2% greater than that of the SSMFs considering the maximum variations. Although this slightly higher variation for the flat fibre compared to that of commercial dosimeter is not significant, having such variation in dosimeters especially for medical application is relatively high. Further insight is required to address the sources of this variation in optical fibres.

The low fading losses of the flat and cylindrical fibre when compared to that of the capillary fibre is to be noted, supported by the fact that in these two media there are proportionately greater TL contributions from deeper defects, sufficient to retain stable memory of the initially absorbed dose. This is implied from the shape of the glow curve for cylindrical and flat fibres as shown in Fig. 5(d,f), where there is a secondary peak appearing at the higher temperature (being most clear in the glow curve for the flat fibre). This represents additional defects that require more elevated excitation temperatures in order to release the trapped electrons from the deep traps, comparison being made against that of capillary fibre. Since a fraction of the trapped electrons in such fibres require higher excitation energy, fading is somewhat less at room temperature compared to the situation for more shallow trapped electrons.

It should be noted that apart from the case of repeatability, the variation observed in TL response and energy dependency of optical fibres are mainly a result of sample preparation and the TL normalization method. As highlighted in the Methods section, all fibre dosimeters in this study are prepared by cutting manually into a length of 5 ± 0.5 mm; thus a variation of up to ±10% (i.e., ~20% variation) is expected from the sample preparation. Additionally, due to very small size, and thereby low weight per fibre sample, the mean mass has been used for normalization, obtained for each fibre type by weighing a group of 10–15 fibre samples, as again presented in the Methods section. These variations can definitely be reduced by accurately cutting the fibre samples into the same length by using an appropriate fibre cleaver. Nevertheless, this does not alter the finding of present study since there is a very large gap between the individual dosimetric performances of the different fibre types. From the dosimeter application point of view, such variation would be still comparable with that of commercially available dosimeters (eg, TLD-100) as we have presented in this paper.

The TL glow curves reveal generation of additional new defects induced due to collapsing of the Ge-doped layer, the deeper traps contributions being greater in the flat fibre. By using CGCD analysis, the original or main peak in all three fibre types is deconvolved into three peaks that are associated with silicon oxygen defect centres (Si-ODCs), Si^¯^ and GeO_2_ and Si and Ge nano-clusters^36^,^39^, the numbers of such defects in the flat fibre being significantly greater than that in the cylindrical followed by the capillary fibre. Deconvolution of the newly generated defect peak has pointed to the higher activation energy of these, clearly being of a different nature of defects.

Thermoluminescence characteristics of three different types of Ge-doped optical fibres i.e., cylindrical, capillary, and flat fibre, all fabricated using a single preform have been demonstrated. The TL response of the flat fibre is far superior to that of the cylindrical and capillary fibres, offering greater sensitivity and uniform response throughout the dose range investigated. The results point to additional defect centres produced in the flat fibre, being mainly due to the fusing of the surface of the interior walls of the capillary fibre. These additional defects in the flat fibre are the basis of the improved TL response. The technique of collapsing fibre surface walls during the fibre drawing process is shown to offer a useful method for creating high sensitivity radiation dose sensors in radiotherapy, not least for *in vivo* dosimetry.

## Methods

**Figure 6:**
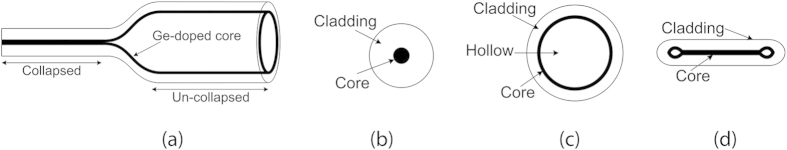
(**a**) Original Ge-doped preform, (**b**) Cylindrical fibre, (**c**) Capillary fibre, and (**d**) Flat fibre.

A Ge-doped core preform has been fabricated using the MCVD process, with ultra-pure fused silica Suprasil F300 glass tube as the substrate. During the MCVD process one part of the preform has been made to collapse to allow for the fabrication of conventional optical fibre, while the other part has been left hollow as shown in Fig. 6a. The preform has then been pulled into conventional optical fibre (hereafter referred to as cylindrical fibre) with 120 μm diameter using the collapsed part (Fig. 6b). The hollow part of the preform has been used to fabricate capillary optical fibre of 140 μm outer diameter and flat fibre with cross section of 60 μm × 180 μm as shown in Fig. 6c,d, respectively. The flat fibre has been fabricated by applying a vacuum pressure of 10 kPa from the top of the hollow preform during the drawing process^40^. Note is made that the capillary fibre in this study is fabricated from the boundary between the collapsed and uncollapsed region of the preform, which leads to smaller hole-to-outer diameter in the capillary compared to the uncollapsed part of the preform. All optical fibres are fabricated within similar range of drawing parameters, i.e., furnace temperature of 1970 ± 20 °C, drawing speed of 1.5–2 m/min, and drawing tension of 30 ± 5 g.

Prior to irradiation, the optical fibres were first cleaned and then manually cut into lengths of around 5 ± 0.5 mm manually using a diamond cone point cleaver. Subsequently, the samples were annealed at 400˚C for 1 hour and then left to cool to room temperature. Five to ten pieces of each fibre type were placed in each of five small plastic bags to allow for delivery of five different radiation doses. The fibres were exposed to photon radiation generated at 6 and 10 MV, with doses of 0.5, 2, 4, 6 and 8 Gy being delivered. For irradiation, the samples were placed at the surface of a *solid water*^*TM*^ phantom (effective atomic number Z_eff_ ~ 7.1, approximately tissue equivalent) with field size of 20 cm × 20 cm and source to skin distance (SSD) of 100 cm. Use of the tissue equivalent phantom, allows direct evaluation of *in-vivo* doses.

After exposing the fibre samples, the TL yield of the fibres were read out using a Harshaw 3500 TLD reader. The time-temperature profile (TTP) was set to a preheat temperature of 50 °C, an acquired temperature rate of 25 °Cs^−1^, an acquisition time of 20 s and a maximum temperature of 400 °C. The mass of the fibre samples were measured using an electronic balance offering accuracy of 0.1 mg. The TL yields were then normalized to the mass of the sample. In this study, the mass of 10–15 fibre samples per species were measured and the mean mass of these were used for normalization.

The analysis of kinetics of thermoluminescence has been carried out through computerized glow curve deconvolution, glow peaks intensity being evaluated with second order kinetics^38^:


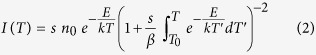


where *s* is the frequency factor of trapped electrons, *n*_0_ is the initial concentration of traps, *E* is the energy of a trap or activation energy (*E*_*a*_) in eV, *T* is temperature in K, *β* is the heating rate and *k* is the Boltzmann constant. The activation energy of each trap is then calculated using the initial rise method^41^.

## Additional Information

**How to cite this article**: Ghomeishi, M. *et al.* Sensitive Fibre-Based Thermoluminescence Detectors for High Resolution *In-Vivo* Dosimetry. *Sci. Rep.* **5**, 13309; doi: 10.1038/srep13309 (2015).

## Figures and Tables

**Table 1:** Minimum detectable dose for TLD samples.

	**α (μC/(mg.Gy))**	MDD(mGy.mg)
Flat fibre detector	0.305 ± 0.040	31 ± 8
Cylindrical fibre detector	0.108 ± 0.013	88 ± 22
Capillary fibre detector	0.057 ± 0.004	167 ± 24
TLD-100	0.480 ± 0.040	20 ± 3

**Table 2:** 

	**Left to Right**	**Peak 1**	**Peak 2**	**Peak 3**	**Peak 4**	**Peak 5**
Cylindrical (a)	*E*_*a*_ (eV)	0.68	1.00	1.19	1.75	2.54
	*s* (s^−1^)	2.95E + 02	1.61E + 05	1.41E + 06	1.92E + 10	6.67E + 15
	*n*_0_ (cm^−3^)	47553	48973	42877	27791	18380
	*T*_max_ (°C)	195	235	283	330	375
	FWHM (°C)	87	69	72	57	42
	Emission wavelength (nm)	1,821	1,240	1,046	710	488
Capillary (b)	*E*_*a*_ (eV)	0.80	1.15	1.38		
	*s* (s^−1^)	2.30E + 04	5.59E + 06	8.22E + 07		
	*n*_0_ (cm^−3^)	25926	43030	24692		
	*T*_max_ (°C)	185	235	283		
	FWHM (°C)	70	60	62		
	Emission wavelength (nm)	1,547	1,078	901		
Flat (c)	*E*_*a*_ (eV)	0.71	0.96	1.37	1.35	1.76
	*s* (s^−1^)	6.46E + 02	1.92E + 04	4.92E + 07	2.07E + 06	3.97E + 08
	*n*_0_ (cm^−3^)	99219	127066	109376	98402	101067
	*T*_max_ (°C)	185	235	283	330	380
	FWHM (°C)	78	74	62	75	47
	Emission wavelength (nm)	1,740	1,295	904	919	703

Trap parameters for: (**a**) cylindrical fibre TLD; (**b**) capillary fibre TLD, and; (**c**) flat fibre TLD. The glow curve deconvolution was performed based on the second order kinetics model of relation (2).
